# Electrospinning of Ultrafine Conducting Polymer Composite Nanofibers with Diameter Less than 70 nm as High Sensitive Gas Sensor

**DOI:** 10.3390/ma11091744

**Published:** 2018-09-17

**Authors:** Qianqian Zhang, Xiaoxiong Wang, Jie Fu, Ruiqiang Liu, Hongwei He, Jianwei Ma, Miao Yu, Seeram Ramakrishna, Yunze Long

**Affiliations:** 1 Collaborative Innovation Center for Nanomaterials & Devices, College of Physics, Qingdao University, Qingdao 266071, China; zhangqianABCABC@126.com (Q.Z.); wangxiaoxiong69@163.com (X.W.); 2017020747@qdu.edu.cn (R.L.); yumiaonju2006@126.com (M.Y.); 2 Industrial Research Institute of Nonwovens & Technical Textiles, College of Textiles & Clothing, Qingdao University, Qingdao 266071, China; hhwpost@163.com (H.H.); 17866827085@163.com (J.M.); 3 Department of Mechanical Engineering, Columbia University, New York, NY 10027, USA; 4 Center for Nanofibers & Nanotechnology, Faculty of Engineering, National University of Singapore, Singapore 119077, Singapore; seeram@nus.edu.sg

**Keywords:** ultrafine fiber, size effect, electrospinning, gas sensor

## Abstract

Polyvinyl alcohol/poly(3,4-ethylenedioxythiophene):poly(styrenesulfonate) (PVA/PEDOT:PSS) composite ultrafine fibers were successfully fabricated by high pressure airflow assisted electrospinning. The electrical properties of PVA/PEDOT:PSS nanofibers with different diameters were characterized. The average diameter of the nanofibers can be down to 68 nm. Due to its large specific surface area, ammonia sensing of the ultrafine nanofibers is more sensitive than the traditional electrospun fibers (average fiber diameter of 263 nm). The ammonia sensing properties of the samples were tested by impedance analysis. The results show that ultrafine PVA/PEDOT:PSS nanofibers are more suitable for detecting low concentrations of ammonia with higher sensitivity.

## 1. Introduction

Electrospinning is an efficient method of preparing long polymer fibers of various properties and diameters (micrometers to nanometers), which enables the direct and continuous preparation of fibers [1,2,3,4,5]. Generally, fibers produced by electrospinning with a diameter of less than 1 μm are referred to as ultrafine nanofibers. However, in a narrow sense, ultrafine nanofibers mean that the fibers have a diameter ranging from 1 to 100 nm. In this paper, improved electrostatic spinning technology can be used to produce true ultrafine fibers with an average diameter of less than 100 nanometers using stronger electrostatic force or auxiliary force. The change of the electrospun fibers’ diameter will cause changes in certain aspects of the properties, such as electrical, magnetic, and mechanical properties, which is called the nano-size effect [6,7,8].

So far, many articles have been published on the use of polypyrrole [9], polythiophene [10], polyaniline [11,12,13], and other conducting polymers to prepare gas sensors. In recent years, poly(3,4-ethylenedioxythiophene)-poly(styrenesulfonate) (PEDOT:PSS) has become an attractive sensing material due to its excellent electrical conductivity and good environmental stability [14]. The PEDOT:PSS/insulating polymer composites also have responsiveness to chemical vapors. For example, electrospun polyvinyl alcohol/PEDOT:PSS (PVA/PEDOT:PSS) fibers and for the production of a gas sensor for detecting formaldehyde and ethanol, and other organic gases are normally measured by the resistance measuring method [15,16,17,18,19]. Electrospun PVA/PEDOT:PSS fibers have high porosity and high specific surface area. Due to these excellent characteristics, sensors based on electrospun fibers can significantly improve the sensitivity and have a faster response time compared to thin-film sensors [20,21,22,23,24].

In this paper, the mass fraction of PVA water solution was 10%, and in order to prepare conductive fibers, PEDOT:PSS conductive water solution was added into the PVA solution. During the high pressure airflow assisted electrospinning experiment, the maximum spinning voltage could be adjusted to 70 kV, and the receiving distance was 120 cm. For comparison, we also prepared PVA/PEDOT:PSS composite fibers as the control group by a traditional electrospinning device (spinning voltage of 20 kV and receiving distance of 15 cm). The aim of this paper was to understand the influence of the specific surface area (size effect) of electrospun fibers on the response characteristics to external environment stimuli [25]. So, for the ultrafine nanofiber membrane and traditional electrospun fiber membrane, we carried out comparative tests to measure and analyze the response characteristics of the PVA/PEDOT:PSS fibers by focusing on the changes in electrical resistance in the ammonia environment. The size effect of electrospun fibers on gas sensing properties such as response time and sensitivity has been observed and discussed.

## 2. Experimental Section

### 2.1. Materials and Methods

PVA (Aldrich, Shanghai, China, MW~67,000) and PEDOT:PSS (1.5% in water, Macklin, Shanghai, China) were used as received. The spinning device is self-assembled by high-voltage power supply (dw-p303-1acfo, Dongwen, Tianjin, China), air blower (kf-8894, Guangdong, China), propulsion Pump (lsp01-1a, Baoding Longer Precision Pump Co., Baoding, China). The morphology and structure of the electrospun fibers were characterized by using a scanning electron microscope (SEM, JSM-6390, JEOL, Tokyo, Japan) with an accelerating voltage of 10 kV. Electrical properties were tested by a Keithley 6487 high resistance meter system (Washington, America) at room temperature and a physical property measurement system (PPMS, Quantum Design, San Diego, CA, USA). In this paper, a RTS-HiR-A 500 mm confocal Raman spectroscopy (Beijing Jin Xianfeng Optoelectronics Technology Co. Ltd., Beijing, China) was used to record the Raman spectra of the samples. The laser emission wavelength was 532 nm.

### 2.2. Preparation of PVA/PEDOT:PSS Ultrafine Nanofibers

**Figure 1:**
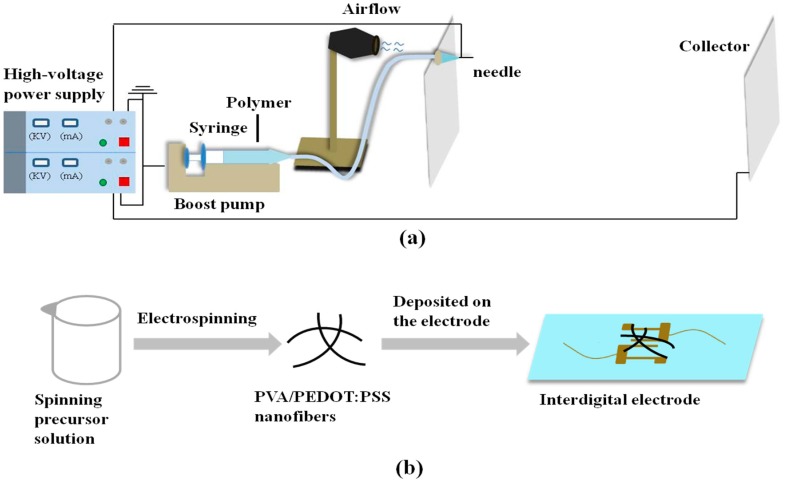
(**a**) Schematic diagram of the experimental device. High voltage between the spinneret and the collector can be increased to 70 kV. The needle and syringe were connected by an extension tube. The distance between the needle and collector was 120 cm; (**b**) schematic illustration of a sensor based on the composite fibers for electrical and gas sensing measurements.

The process of preparing a spinning precursor solution is as follows. PEDOT:PSS conductive liquid was added into PVA solution to obtain a 20 wt % solution. Specifically, 5 g of PVA powder was added into 35 g of water and was stirred for 4 h to form a transparent homogeneous PVA solution. Then, 10 g of PEDOT:PSS solution was mixed into the above solution and was stirred for 2 h to obtain a homogeneous solution. A schematic diagram of the spinning device is shown in Figure 1a. A negative 35 kV voltage was applied to the nozzle and a positive 35 kV voltage was applied to the collector, so a typical 70 kV voltage difference was applied between the nozzle and the collector. The distance from the spinneret to the collector (spinning distance) was 120 cm. During the experiment, the airflow was used to assist in spinning and collecting the fibers, and the wind speed was set at 10 m·s^−1^. Through the modified device that is shown in Figure 1a, we prepared the ultrafine nanofiber membrane. For comparison, we also electrospun the PVA/PEDOT:PSS fiber membrane with the traditional electrospinning device (voltage 20 kV and receiving distance 15 cm) as the control group. The ratio of the two solutions was exactly the same.

## 3. Results and Discussion

### 3.1. Characterization of Morphology and Structures 

**Figure 2:**
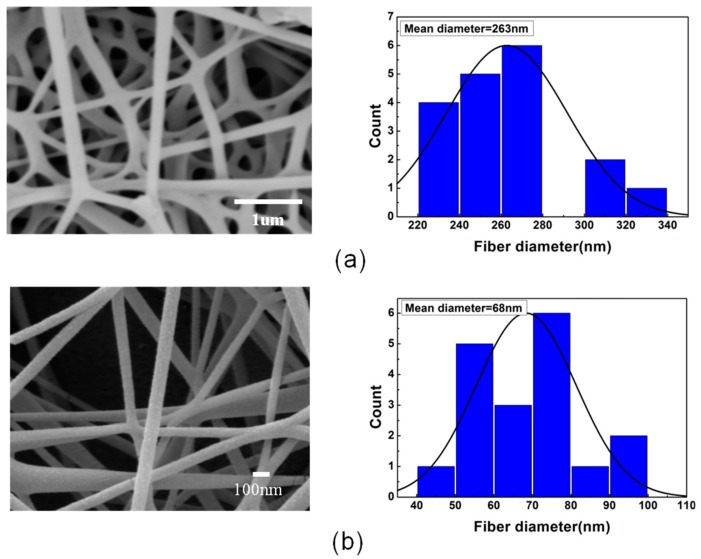
SEM images of PVA/PEDOT:PSS fabricated under (**a**) traditional electrospinning (20 kV), (**b**) high pressure airflow assisted electrospinning (70 kV), and the fiber diameter distribution table of each group of fibers is shown on the right.

For the conventional spinning device, the electrospun PVA/PEDOT:PSS fibers had an average diameter of 250–300 nm. In this work, the average diameters of the PVA/PEDOT:PSS composite fibers decreased with increasing voltage. We measured the diameter of the electrospun fibers under different voltages. In the 20 kV condition, the spinning distance was set to 15 cm as a conventional electrospinning experiment. The other spinning distance was set to 120 cm, and the wind speed remained unchanged at 10 m·s^−1^. The SEM (scanning electron microscope) images show that as the spinning voltage increases, the average fiber diameter decreases (see Figure 2). As can be seen from the particle size analysis, under conventional spinning conditions (voltage 20 kV and collecting distance 15 cm), the fibers are relatively coarse with an average diameter of 263 nm. With the voltage increasing and the spinning distance increasing, the average diameter of the PVA/PEDOT:PSS nanofibers could be decreased to 68 nm.

### 3.2. Raman Spectrum Analysis

**Figure 3:**
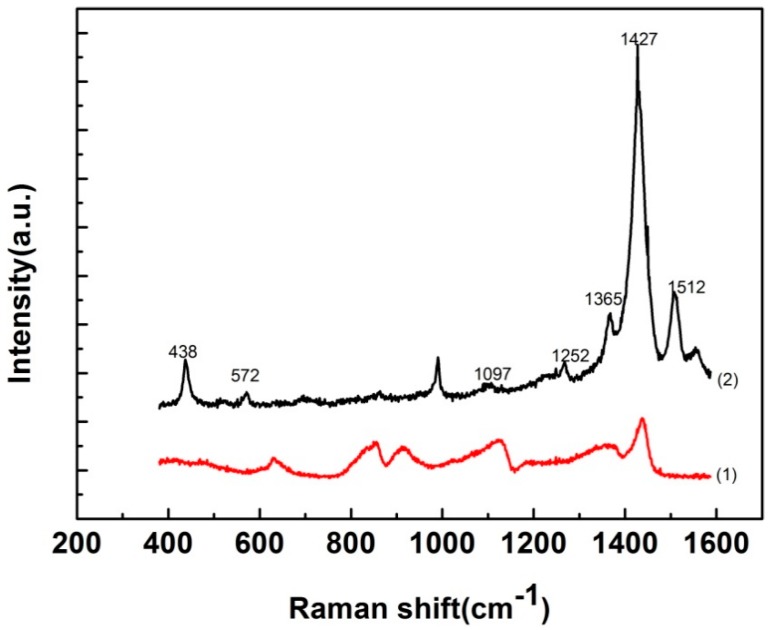
Raman spectra of the (1) electrospun pure PVA fibers, (2) PVA/PEDOT:PSS composite fibers.

It is well known that Raman spectroscopy is a powerful tool for studying conductive polymers. The resultant PVA/PEDOT:PSS composite fibers were characterized by Raman spectrum analysis. Figure 3 shows the Raman spectrum of the as-prepared samples. (1) represents the pure PVA Raman shift peaks. Its characteristic peak was relatively weak, including 854 cm^−1^ (γ(CC), skeletal), 1110 cm^−1^ (stretch(CO)) and 1441 cm^−1^ (δ(CH_2_), O-SO_2_-O). (2) represents the Raman shift peaks of the PVA/PEDOT:PSS composite fibers. Typical representative peaks of the PVA/PEDOT:PSS composite fibers were at 438 cm^−1^ (SO_2_ bending), 572 cm^−1^ (oxyethylene ring deformation), 1097 cm^−1^ (the C-O-C deformation), 1252 cm^−1^ (C-C interring stretching), 1365 cm^−1^ (C-C stretching), 1427 cm^−1^ (the symmetric C=C(-O) stretching) and 1512 cm^−1^ (the asymmetric C=C stretching). The observed band position and distribution are consistent with the published data [26,27,28,29]. Compared with the Raman shift peaks of the electrospun PVA fibers, we can infer that PEDOT:PSS was successfully doped into PVA fibers.

### 3.3. Electrical and Gas Sensing Measurement

**Figure 4:**
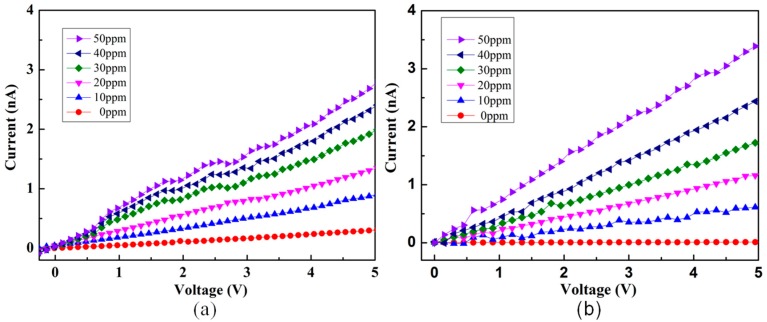
I-V curves of the two groups of fiber membranes measured under different ammonia concentrations. (**a**) Conventional fibers electrospun under 20 kV (average fiber diameter of 263 nm), (**b**) ultrafine fibers electrospun under 70 kV (average fiber diameter of 68 nm). The ammonia concentration increased from 0 ppm to 50 ppm.

**Figure 5:**
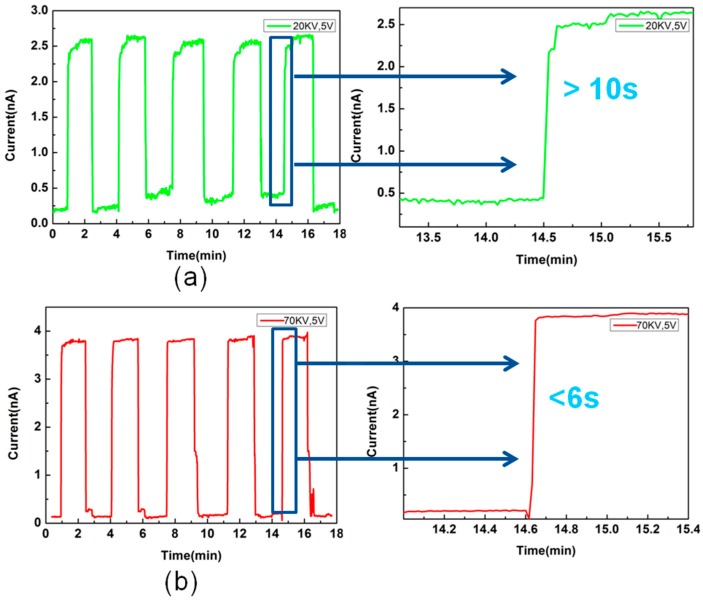
Reversible response curves of current-time (I-t) to NH_3_ concentration of 50 ppm. (**a**) The graph is a 5-cycle test of a conventional fiber membrane electrospun under 20 kV (average fiber diameter of 263 nm) with a response time greater than 10 s. (**b**) The graph is a series of 5 cycles of an ultrafine fiber membrane electrospun under 70 kV (average fiber diameter of 68 nm) with a response time of less than 6 s. The test voltage was 5 V.

We made the interdigital electrode by spraying gold on the slide glass as a supporting part of the fiber membrane sensor and connected the measuring instrument with copper wire at both ends of the interdigital electrode. We then attached a suitable fibrous membrane to the interdigital electrode, as shown in Figure 1b. The electrical and NH_3_ sensing properties of the sample were measured by the Keithley 6487 high-resistivity instrument and the homemade gas sensor system under a constant temperature (20 °C). The electrical data was recorded automatically by a computer. Figure 4 shows the current-voltage (I-V) curves of the PVA/PEDOT:PSS ultrafine fibers that were electrospun under 70 kV with different ammonia concentrations to compare the effect on the electrical properties due to the size effect of the fibers. The I-V curve of the conventional PVA/PEDOT:PSS fibers that were electrospun under 20 kV was also characterized. The Keithley 6487 is a famous small current testing instrument that can measure very weak current. Its measurement limit is 10^−13^ A, while the current in our test process was 10^−9^ A, which can fully meet the test requirements. Due to the lack of good electromagnetic shielding, electromagnetic interference was inevitable during the test. Therefore, the curve we tested had slight pulsations, however these pulsations were consistent with the gaussian distribution, which did not affect the overall linear trend. Our linear trend conforms to the typical linear I-V curve, so it can reflect the true state of the sample. From the I-V curves shown in the figure, we can see that with the ammonia concentration increasing, the current was proportional to the voltage. In addition, it can also be seen that in the absence of ammonia, the ultrafine fiber sample was less conductive than the conventional fiber sample. In addition, these two samples were also tested for I-t (current-time) cycling at 50 ppm ammonia concentration, and their sensitivity was compared by comparing their response time to ammonia. As shown in Figure 5, the reversible responses of the PVA/PEDOT:PSS fibers at 50 ppm concentration were tested. Two sets of samples were tested for 5 cycles respectively. Their sensitivity to ammonia based on their response time to the ammonia were compared. The voltage applied during the test was 5 V. When the given concentration of NH_3_ was pumped into the container, the resistance of the sample increased quickly. When the NH_3_ was pumped out, the decline of the resistance was also fast. Figure 5a shows that the response time of the conventional fiber membrane was greater than 10 s at a 50 ppm NH_3_ concentration and that the ultrafine fiber membrane in Figure 5b was less than 6 s. Comparing (a) with (b) in Figure 5, it can be found that the ultrafine fibers (average fiber diameter of 68 nm) have shorter response times under low-concentration ammonia than the traditional electrospun fibers (average fiber diameter of 263 nm). Namely, the response speed of the ultrafine fibers is faster.

**Figure 6:**
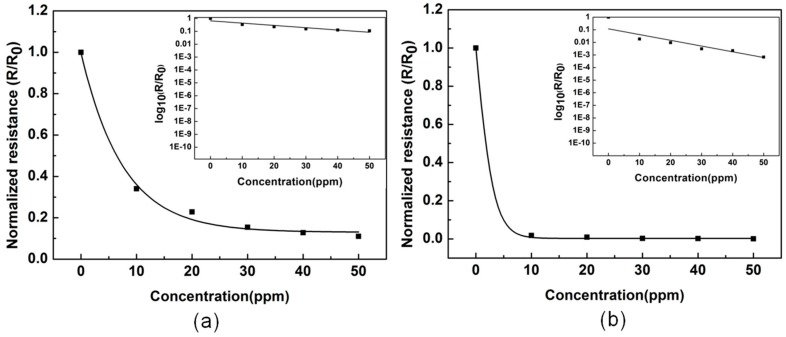
Resistance changes of (**a**) conventional electrospun nanofiber film with an average fiber diameter of 263 nm and (**b**) ultrafine nanofiber film with an average fiber diameter of 68 nm upon exposure to different concentrations of ammonia. R/R_0_ is the resistance (R) normalized to the initial resistance (R_0_) prior to gas exposure.

The corresponding resistances of each concentration are shown in Figure 6, which shows that the observed resistance decreases with concentrations of ammonia. The resistance change curve of a conventional electrospun fiber membrane is shown in Figure 6a, and Figure 6b represents the resistance change curve of the ultrafine nanofiber membrane. Through the comparison of (a) and (b), we can see that the resistance of the ultrafine fibers is much larger than the conventional fibers in low concentrations of ammonia. It also shows that ultrafine fibers are more sensitive to this change in concentration at low ammonia concentrations. This may be due to the small diameter of the ultrafine nanofibers causing the high surface area within the film to be rapidly approached by the gas. This figure shows that fine diameter fibers are more suitable for detecting low concentrations of ammonia with higher sensitivity. Therefore, the ultrafine fibers seem to have better performance in terms of sensitivity and time response than conventional fibers in low concentrations of ammonia.

**Figure 7:**
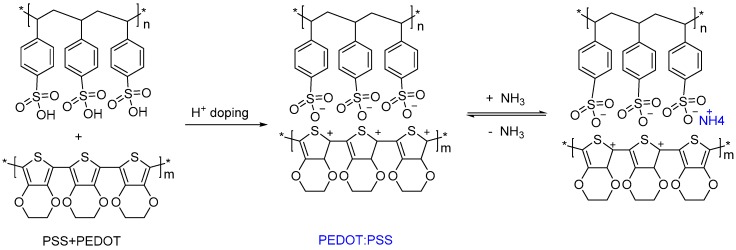
Diagram of the reaction mechanism of PEDOT:PSS and ammonia gas.

According to previous reports, the sensing mechanism of PVA/PEDOT:PSS composite nanofibers that were obtained in this paper can explain that PEDOT:PSS is conductive because the hydrogen protons in PSS are doped into PEDOT to make it conductive. The detection of NH_3_ occurs via the mechanism that is shown in Figure 7, which shows the interaction between NH_3_ and PEDOT:PSS. This process is a reversible reaction process. NH_3_ was absorbed during the reaction and NH_4_^+^ was generated by the neutralization of hydrogen protons. Meanwhile, the oxidation state of PEDOT changed (called the dedoping effect) and then the conductivity decreased. Although this interaction can reduce the carriers and increase the resistance, the overall fiber does not show the characteristics of PEDOT due to the low doping degree of it (20 wt %) and the conductance of the sample is very poor. On the one hand, water molecules in mixed gas can improve the contact between fibers and reduce the contact resistance, thus improving the conductivity of fiber samples [30]. Moreover, PVA is a water soluble polymer, the composite PVA fibers can absorb water molecules and ammonia gas, and the ammonia gas disassociates into ammonium ion and hydroxide ion, which will also improve the electrical conductivity of the composite PVA fibers. As the latter mechanism plays a leading role, the overall conductance of the composite fibers increases with the increase of ammonia concentration.

## 4. Conclusions

In summary, PVA/PEDOT:PSS ultrafine nanofibers with an average fiber diameter of 68 nm were successfully fabricated via electrospinning and were prepared as a sensor for ammonia sensing. The size effect of the electrospun fiber diameter on its sensing properties were observed. The ultrafine nanofibers appeared to have better performance in time response compared to the conventional electrospun fibers (with a fiber diameter of 263 nm) in the detection of low ammonia concentrations. Through the investigation and research, we summarized the research situation of the ammonia sensor based on nanomaterials as shown in Table 1. The results show that ultrafine composite nanofibers are more suitable for detecting low concentrations of ammonia with higher sensitivity. In addition, the reduction of the fiber size greatly improved the fiber sensing performance, which means that it can be widely used in electronics, gas sensing, and so on.

## Figures and Tables

**Table 1:** Sensors based on nanomaterials detects ammonia.

Materials	Array of Fibers	Diameter	Operating Temperature (°C)	Detection Limit
PANI [31]	-	300 nm	RT	92 ppm
PAA-PVA [32]	Nonwoven	100–400 nm	RT	50 ppm
PANI [33]	-	-	RT	100 ppm
Graphene/PEDOT:PSS [34]	-	-	RT	5 ppm
WO_3_ [35]	Nonwoven	20–140 nm	350 °C	50 ppm
PEDOT [36]	nanowire	350 nm	RT	10 ppm
PANI [37]	-	-	RT	50 ppm
PEDOT [38]	nanotube	140 nm	RT	5 ppm
Palladium/polypyrrole [39]	-	15–35 nm	RT	20 ppm
PANI/TiO_2_ [40]	-	-	RT	23 ppm
